# Advances in artificial intelligence in thyroid-associated ophthalmopathy

**DOI:** 10.3389/fendo.2024.1356055

**Published:** 2024-04-23

**Authors:** Chenyuan Yi, Geng Niu, Yinghuai Zhang, Jing Rao, Guiqin Liu, Weihua Yang, XingZhen Fei

**Affiliations:** ^1^ Guangdong Key Laboratory of Biomedical Measurements and Ultrasound Imaging, School of Biomedical Engineering, Shenzhen University Medical School, Shenzhen, China; ^2^ School of Medical Technology and Nursing, Shenzhen Polytechnic University, Shenzhen, China; ^3^ Shenzhen Eye Institute, Shenzhen Eye Hospital, Jinan University, Shenzhen, China; ^4^ Department of Endocrinology, First People’s Hospital of Huzhou, Huzhou University, Huzhou, China

**Keywords:** artificial intelligence, deep learning, thyroid-associated ophthalmopathy, diagnosis, treatment

## Abstract

Thyroid-associated ophthalmopathy (TAO), also referred to as Graves’ ophthalmopathy, is a medical condition wherein ocular complications arise due to autoimmune thyroid illness. The diagnosis of TAO, reliant on imaging, typical ocular symptoms, and abnormalities in thyroid function or thyroid-associated antibodies, is generally graded and staged. In recent years, Artificial intelligence(AI), particularly deep learning(DL) technology, has gained widespread use in the diagnosis and treatment of ophthalmic diseases. This paper presents a discussion on specific studies involving AI, specifically DL, in the context of TAO, highlighting their applications in TAO diagnosis, staging, grading, and treatment decisions. Additionally, it addresses certain limitations in AI research on TAO and potential future directions for the field.

## 1 Introduction

### 1.1 A brief history of artificial intelligence and its evolution

The genesis of Artificial Intelligence (AI) can be traced back to the mid-20th century, specifically to the 1950s (1), heralding a seminal epoch in the domain of computer science. Initial forays into AI were primarily concerned with the replication of elementary human cognitive abilities, encompassing problem-solving and algebraic computations. These endeavors laid the groundwork for what would burgeon into a diverse and expansive field. Subsequent developments saw AI research extend its ambit to encompass pattern recognition, natural language processing, and knowledge representation, thereby illustrating the field’s adaptability and its capacity to address a wide spectrum of applications.

The 1970s witnessed a paradigmatic shift with the advent of Machine Learning (ML), a distinct subset within AI characterized by data-driven algorithms (2). This period was marked by seminal contributions, such as those by Clark et al., who leveraged pattern recognition methodologies for the classification of crude oil gas chromatograms, achieving unprecedented accuracy. Such advancements underscored the potential of ML to revolutionize practical applications. Concurrently, the investigations by Cui et al. into the deployment of ML for the enhancement of biosensors delineated a pivotal shift in AI research, highlighting ML’s transformative potential within the broader AI landscape (3).

The advent of Deep Learning (DL), a sophisticated offshoot of ML, has in recent years catalyzed a significant revolution within the field (4). Characterized by its utilization of intricate neural networks, DL has facilitated major breakthroughs, particularly in the realms of image and speech recognition. Notably, Alani’s work, which achieved a 98.59% accuracy rate in recognizing Arabic handwritten digits through DL, serves as a testament to the efficacy of this approach. Within the DL paradigm, Convolutional Neural Networks (CNNs) are distinguished by their specialized layer architecture, tailored for image-centric tasks. The efficacy of CNNs has been demonstrably proven across a spectrum of computer vision applications, ranging from image classification to facial recognition (5–7), thereby epitomizing the depth and sophistication of contemporary AI methodologies.

This succinct historical exposition not only chronicles the evolutionary trajectory of AI from its nascent stages, aimed at mimicking rudimentary cognitive functions, to its present stature as a cornerstone of technological innovation but also delineates the hierarchical relationship amongst AI, ML and DL. This hierarchical structure, wherein AI encompasses ML as a strategy for achieving artificial intelligence, with DL further refining ML through advanced neural network architectures, reflects the incremental complexity and the expanding problem-solving capabilities of the field.

### 1.2 Thyroid-associated ophthalmopathy

Thyroid-associated ophthalmopathy (TAO), also known as Graves’ ophthalmopathy, is an ocular complication linked to autoimmune thyroid disease. It is closely linked to Graves’ disease, with clinical relevance in about 50% of patients, but it may also arise from other disorders of abnormal thyroid function. Thyroid-associated ophthalmopathy (TAO) has a high prevalence among individuals aged 50-60 and 70-80 years, and is more common in women with a 1:4 ratio of women to men in the more severe forms of ophthalmic disease (1). The main symptoms of TAO include exophthalmos, eyelid swelling, double vision, eye pain, and visual impairment. The disease’s pathogenesis is not fully understood, but studies have shown that an autoimmune response is an essential factor in its development (2). The diagnosis of TAO relies on objective criteria, including typical ocular symptoms, abnormalities in thyroid function or thyroid-related antibodies, and imaging findings. Following diagnosis, clinicians typically stage and grade the disease according to its level of activity. During the active phase, patients experience symptoms of ocular redness and swelling, pain, and increased lacrimation due to inflammation. In the inactive phase, inflammatory symptoms decrease or disappear, but structural changes like exophthalmos and ocular muscle dysfunction may still be present. Disease severity is evaluated based on the grading system. Mild thyroid-associated ophthalmopathy (TAO) is characterized by mild eyelid erythema and swelling. Moderate TAO may be accompanied by exophthalmos and double vision in addition to the aforementioned symptoms. Severe TAO may result in optic nerve compression, severe proptosis, and visual impairment (3).

### 1.3 AI in ophthalmology and its potential applications

Since the beginning of the 21st century, there has been a gradual increase in the use of ML and DL in healthcare. This rise can be attributed to the increased computing power and the prevalence of Big Data. These algorithms are commonly utilized for various tasks such as disease prediction (4), diagnostic assistance (5) and medical image analysis (6) and many other tasks. Especially in medical image analysis, DL techniques like CNN demonstrate reliable and accurate processing of complex image data including MRI, CT and X-rays (7–9).

Ophthalmology relies heavily on image-based diagnostics. In recent years, AI, particularly DL techniques, has found widespread application in ophthalmic diseases, encompassing retinal disease diagnosis, corneal morphology analysis and glaucoma detection (10–12). Yang et al. (13) synthesized numerous clinical evaluations of AI research and established a clinical research evaluation guide for ophthalmic AI. For TAO, AI presents significant potential for both research and application. TAO is a complex immune system-related disease with an unclear etiology and mechanism. Yet, AI technology enables the extraction of meaningful patient data for more accurate diagnoses and progress assessments by physicians. Furthermore, DL models can aid in analyzing medical images, such as eye MRI, CT scans, photographs of the external eye, and other visuals, to offer TAO patients personalized and more accurate treatment recommendations.

In this summary, we explore the historical progression of AI, tracing its development from rudimentary cognitive simulations to recent advances in DL. Additionally, we provide an overview of Thyroid-associated ophthalmopathy (TAO), detailing its clinical characteristics, pathogenesis, and staging and grading. We highlight the significant role of AI in ophthalmology, particularly in its potential to diagnose and treat TAO, emphasizing its vital impact in the field. In this article, we will examine four components of AI in clinically diagnosing, staging, grading, and treating TAO to provide a comprehensive insight into AI’s potential in the TAO field.

## 2 Role of AI in TAO diagnosis

### 2.1 TAO diagnosis

The diagnosis of TAO relies on three major factors (1): ocular symptoms that are typical, for example Enlarged Extraocular Muscles, Strabismus, and Diplopia (2); abnormalities related to thyroid function or thyroid antibodies; and (3) imaging indications, such as Enlarged Extraocular Muscles (3). Huang et al. (14) Explored the importance of ophthalmic characteristics in detecting thyroid-associated ophthalmopathy and proposed that TAO subtypes, stages, and severity can be identified by utilizing auxiliary references, such as demographic factors, complaint symptoms, and image features. These non-intrusive markers can be administered promptly for clinical evaluation of TAO status.

Traditional diagnosis of TAO may rely on subjective clinical assessment and interpretation, leading to inconsistent diagnostic results among physicians and inaccuracies. Detecting TAO at an early stage is also challenging, resulting in delayed appropriate treatment and management. Traditional methods are also time-consuming as a single diagnosis often necessitates complex radiological examination and multiple evaluations, resulting in inefficient diagnoses.

With the advancement of AI technology, the use of DL in the medical field is becoming increasingly prevalent. As a current prominent area of AI research, DL can process vast amounts of diverse data, such as images, videos, and text, allowing for large quantities of clinical and imaging data to be utilized to enhance the precision and consistency of TAO diagnoses. Through automated image analysis and data processing, DL can provide timely or rapid diagnostic feedback, expediting the diagnostic process and enabling early intervention possibilities.

### 2.2 AI in TAO diagnosis

Eyelid abnormalities, mainly characterized by the retraction of the upper and lower eyelids, are a prevalent manifestation of Thyroid-associated Ophthalmopathy (TAO). Precise eyelid measurement is vital for TAO diagnosis, severity grading, surgical planning, and treatment outcome evaluation. Clinicians traditionally assess eyelid position in TAO by manual measurement with a ruler. The study concentrates on one-dimensional characteristics, specifically Palpebral Fissure Length and Margin Reflex Distance (15). Manual measurement of precise eyelid parameters necessitates extensive expertise on the clinician’s part and the patient’s cooperation. Follow-up measurements are additionally difficult to perform continuously and consistently due to inter-observer variations. Obtaining standardized and precise measurements of eyelid characteristics is crucial to enhance the diagnosis and treatment of TAO.

Shao et al. (16), a DL network can be employed to detect and segment the eye, comprising two stages: first identifying the eye and subsequently segmenting the Eyelid and Corneal Limbus. By converting eyelid parameters into actual distances, this method demonstrated strong agreement (correlation coefficients of 0.932 to 0.980) when compared with measurements obtained by professional ophthalmologists. Additionally, this method provided comprehensive measurements in just 3 seconds.

After exploring the effective use of DL in accurately measuring eyelid abnormalities, we shift our focus to measuring exophthalmos. AI techniques have also demonstrated their potential in addressing another critical symptom of TAO. exophthalmos, also known as proptosis, is a medical condition where the eyeball bulges out from the orbit. This condition could be caused by structural changes within the eye socket or by a factor behind it. exophthalmos can manifest as either bilateral or unilateral, and its severity spans from mild to significant protrusion (3). The degree of severity can fluctuate from mild to substantial. exophthalmos protrusion is clinically diagnosed by observing and measuring its degree, typically with the Hertel exophthalmoscope (17). Although the Hertel Proptosis Meter is the most widely employed method, it has the drawback that the operator or measurement environment may affect the findings, resulting in inadequate reproducibility, reduced reliability, and high subjectivity. In radiological examination, computed tomography (CT) provides precise imaging of the orbit and eyeball, enabling accurate assessment of the extent of exophthalmos. Additionally, two-dimensional and three-dimensional images make it possible to obtain measurements in different planes with reduced human error and subjective judgment compared to traditional proptometers. CT scans have thus become the primary means of examining exophthalmos. However, it is still impossible to eliminate completely the effects of human error and subjective judgment. By utilizing DL technology, exophthalmos can be automatically measured, enhancing the accuracy and reliability of the measurement. This approach offers the clinicians a more precise and unbiased diagnostic basis.

Zhang et al. (18) conducted an analysis of orbital CT images utilizing DL to measure the degree of exophthalmos. Results demonstrated that their model produced consistent measurements with clinician’s results, with a correlation coefficient of 0.9902, and took less than one second to analyze each image. Technical language was used when necessary, and all technical abbreviations were explained in the text. Additionally, the writing maintained a formal, objective tone. Demonstrating its efficiency and accuracy. Fu et al. (19) used a CNN to automatically identify the exophthalmos and the posterior crypt region of the eye in CT images and proposed two metrics: linear displacement and volumetric displacement to quantify the degree of exophthalmos prominence. This study is pioneering in the use of computer software to quantify volumetric exophthalmos protrusion, improving surgical prediction accuracy. Additionally, it showcases the potential of DL technology in calculating the volume of Inferior Orbital Abscesses.

After investigating the effectiveness of DL in measuring exophthalmos, we are now expanding our research to explore a wider range of AI applications. In their studies, Song et al. (20) collected 1,435 CT images, then trained and validated the 3D-ResNet model. In external validation, the model demonstrated high accuracy with an AUC of 0.919 and performed comparably to the resident group in a noninferiority experiment. The application of this model has undergone testing and conforms to the regulations of a clinical trial, indicating a technological foundation for the automated diagnosis of TAO on orbital CTs. This screening model enables the identification of patients displaying relevant characteristics on CT scans to achieve a comprehensive and precise diagnosis.

Huang et al. (21) presented an extensive framework demonstrating the potential application of AI in diagnosing ocular symptoms in TAO. Their novel AI-based system utilizes facial images to detect ocular symptoms and is divided into three modules, with the aim of identifying eye position, ocular motility disorders, and related signs. A sample of 21,840 images was obtained from 1,560 patients (3,120 eyes). The system attained a mean AUC of 0.91 for the seven symptoms in the test dataset, demonstrating superior performance.

In conclusion, the advanced AI and DL technologies have increased efficiency, accuracy, and reliability in TAO screening and diagnosis when compared to conventional methods. This chapter emphasizes the potential value of AI technologies in TAO diagnosis by demonstrating how DL technologies can assist physicians in making more precise diagnostic and treatment decisions. Researches on AI in TAO diagnosis are shown in **Table 1**.

**Table 1:** AI in TAO diagnosis.

Authors (Year)	Task	Input Data Type	Samples Dataset	Model	Metrics
Shao et al. (22)	Detecting and segmenting the eye for eyelid measurement	Eye images	148	Attention R2U-Net	Correlation coefficients of 0.932 to 0.980
Zhang et al. (23)	Measuring the degree of exophthalmos in orbital CT images	Orbital CT images	178	U-Net	Correlation coefficient of 0.9902
Fu et al. (24)	Identifying exophthalmos and quantifying its degree	CT images	56	CNN	Linear and volumetric displacement
Song et al. (25)	Automated diagnosis of TAO on orbital CTs	CT images	1435	3D-ResNet	AUC of 0.919
Huang et al. (26)	Diagnosing ocular symptoms in TAO	Facial images	21840	AI-based system	Mean AUC of 0.91

## 3 Role of AI in TAO staging

### 3.1 TAO staging

TAO is an autoimmune inflammatory reaction specific to the organs, divided into active and inactive phases. Patients may experience the active phase of the disease for 18-24 months, followed by a gradual transition to the inactive phase (27). It is important to note that this information has been cited as a reliable source. Staging TAO enables treatment planning, surgical timing selection, and prognosis assessment. Clinicians commonly stage the disease activity of patients with a first-time TAO diagnosis using the clinical activity score (CAS) as the initial measure. Clinicians commonly stage the disease activity of patients with a first-time TAO diagnosis using the clinical activity score (CAS) as the initial measure (22). The CAS score comprises seven elements, namely retrobulbar pain that occurs spontaneously, pain experienced during eye movement, eyelid congestion, eyelid edema, Conjunctival Prominence, Eyelid Swelling, and Lacrimal Prominence Swelling. The score is rated at 1. An active stage is classified when the CAS is ≥3, whereas the inactive stage when CAS <3. These include an increase in exophthalmos of 2 mm or more, a decrease in eye movement by 8° or more (as determined by Goldmann visual field meter or synoptic examination result), and a decrease in visual acuity by 1 line or more. A follow-up CAS score of ≥4 out of a total score of 10 was indicative of a state of activity. When utilizing the Clinical Activity Score (CAS) to monitor and evaluate the effectiveness of TAO treatment, it is necessary to incorporate an additional 3 criteria beyond the standard 7. The combination of orbital MRI findings, as well as the CAS, was subsequently utilized to determine staging.

An orbital MRI that shows a high signal on the T2-weighted image (T2WI) of the extraocular muscles suggests an active stage of TAO (28), a complex autoimmune disorder characterized by cosmetic damage and vision loss. On the other hand, the absence of signal intensity change implies an inactive stage. An enlarged lacrimal gland with increased signal intensity also indicates an active phase. The application of quantitative MRI (23–25), a pivotal diagnostic approach in TAO, provides precise demonstrations of orbital lesions and a deeper understanding of disease conditions through detailed morphological and functional analyses. Although the fast evolution of MRI techniques and TAO’s complexity pose challenges, the integration of quantitative MRI enhances clinical decision-making in TAO management. Multimodal MRI’s quantitative parameters are clinically valuable in TAO staging (3). Thus, the diverse quantitative parameters of multimodal MRI, including CAS which is quick to score, become invaluable in accurately staging TAO and facilitating its treatment. This streamlined approach to utilizing quantitative MRI underscores the need for bridging clinical and radiological insights, promising improved outcomes in the multidisciplinary management of TAO.

However, computer-assisted diagnosis systems may be affected by subjective factors from both examiners and examinees, leading to biased results (26). In addition, conventional staging methods often depend on clinicians’ experience and subjective judgment, creating potential subjectivity and inconsistency. Because traditional methods entail multiple examinations and evaluations, they result in increased diagnostic and staging delays, thereby delaying patients’ access to timely treatment. Possibly due to limitations in technology and knowledge, conventional staging methods may not provide an accurate assessment of the severity of the condition and the extent of the lesions.

AI can yield more consistent and unbiased diagnostic results via algorithmic and big data analyses, minimizing human error. This technology can also rapidly process large datasets to facilitate swift and efficient disease diagnosis and staging, leading to cost savings in healthcare. DL and ML algorithms can enhance the precision of diagnosing and staging Thyroid-associated ophthalmopathy. This can be accomplished by utilizing extensive training data to attain early diagnosis and staging. Early detection enables timely treatment measures, thereby improving patient prognosis. Large-scale automated screening can also be implemented, especially in large populations, to promptly detect diseases and increase the detection rate.

### 3.2 Application of AI in TAO staging

In recent years, multiple studies have corroborated the effectiveness of AI-based TAO staging techniques.

It should be noted that CAS outcomes may vary based on the evaluator, and an experienced ophthalmologist is necessary to ensure precise assessments. Moon et al. (29) have created a ML-supported system that assists in evaluating five CAS symptoms in patient facial images and forecasts CAS by taking into account two subjective symptoms. The algorithm was trained using 1020 patient facial images with TAO. Ultimately, the system’s ability to detect the early stages of TAO based on CAS was demonstrated through tests using a consistent dataset provided by three ophthalmologists. The sensitivity and specificity for diagnosing active TAO were 0.881 and 0.869, respectively. Additionally, the diagnostic accuracy AUC for each inflammatory sign of CAS ranged from 0.884 to 0.977. These results indicate the potential utility of the system for early detection before any visible changes in appearance occur.

Since orbital MRI offers deep features for TAO clinical staging, it can serve as a valuable tool for TAO staging during Radiological Examination. Lin et al. (28) conducted a study, collecting and analyzing 160 MRI images, and developed a DL system based on CNN that effectively distinguishes between active and inactive patients. The system achieved an accuracy of 0.86, a sensitivity of 0.82, a specificity of 0.89, and an F1 score of 0. 71. The study shows that the network developed by Li et al. (30) performed well in evaluating the inflammatory activity stage of extraocular muscles in TAO. The ML models were built using contrast-enhanced MRI images from 1479 TAO patients, and the best-performing model achieved an AUC of 0.9260, precision of 0.9118, recall of 0.9609, and F1 score of 0.9357 in the test set. The activity assessment model (TAO) developed in this study boasts excellent classification efficiency and affordability, thus making it ideal for seamless adoption in community hospitals.

CT scans provide detailed images of the orbit and surrounding structures, aiding physicians in understanding TAO stages. Lee et al. (31) introduced a neural network technique that utilizes orbital CT images of 144 active TAO patients and 288 inactive TAO patients as data, achieving a final AUC of 0.871 on an internal test set. Their findings demonstrate that DL, coupled with CT images, can differentiate between patients with active and inactive stages of TAO.

Compared to using MRI and CT images, utilizing TAO facial RGB images for ocular lesion characterization and TAO staging is a significant and cost-effective method for healthcare providers. Zhu et al. (32) developed an EDWM model by training it on 2,228 facial images of TAO patients, resulting in accurate recognition of TAO. On a test set of 600 facial images, the EDWM model achieved a Dice coefficient of 0.898, sensitivity of 0.895, and precision of 0.931. These results establish a more dependable and straightforward diagnostic foundation for detecting TAO early and acquiring CAS scores.

Single-Photon Emission Computed Tomography(SPECT)/CT merges SPECT’s functional imaging with CT’s anatomical imaging, providing additional details about TAO staging and precise lesion localization in the orbital region. Yao et al. (33) introduced the automated GO-Net method for detecting inflammatory activity in TAO patients. The study is partitioned into two sections, the SV-Net segmentation network, and the CNN based on SPECT/CT images. Following their evaluation on a test set, the outcomes of the segmentation task produced 0.82 ± 0.05%, 91.01 ± 5.32%, and 99.96 ± 0.03% for IOU, SN, and SP, correspondingly. This indicates the segmentation model’s promising performance. For predicting active GO, the classification model exhibited optimal results with an AUC of 0.90 and a diagnostic precision of 86.10%. This indicates that the method has good accuracy and consistency in the determination of TAO staging.

### 3.3 New AI methods that can be used for TAO staging

In the general clinical practice, there is sufficient ophthalmological data and demographic information available to efficiently acquire multiple non-invasive ophthalmic images in a timely manner. These images may assist in the supplementary diagnosis of various subtype features of TAO. For changes in thyroid function, stage, and severity, it is valuable to investigate the clinical significance of demographic and ophthalmogram features for the diagnosis and assessment of TAO prior to undergoing biochemical index testing, despite the existence of various TAO-recognizing biomarkers. A retrospective study by Huang et al (14). encompassing over 1,000 medical records (with a total of 953 patients) and ML models resulted in several feasible regression models that could aid in TAO staging. Ultimately, demographic factors, complaint symptoms, and image features (including lid congestion, Conjunctival Hyperemia, and Corneal Ulcer) can predict TAO subtype, stage, and severity.

Due to changes induced by disease in the appearance of the eyes and face, as well as disruptions to mental well-being, patients with TAO exhibit distinctive facial expressions that deviate from those of healthy individuals. Facial expressions can reflect both the objective facial deformities and subjective psychological disturbances of patients, and clinical activities, disease severity, and quality of life (34). Lei et al. (35) utilized the VGG-19 network to automatically classify facial expressions using two datasets: 827 cases of data in dataset 1 and 126 cases of data in dataset 2. The model achieved an AUC of 0.847, an accuracy of 0.851, a sensitivity of 0.899, a precision of 0.899, a specificity of 0.720, and an F1 score of 0.899 on the internal test set. The researchers investigated the relationship between facial expression and clinical activity, disease severity, and quality of life. Their findings demonstrated a significant association between facial expression and all three factors. This adds a new dimension to the staging of TAO. Researches on AI in TAO staging are detailed in **Table 2**.

**Table 2:** AI in TAO staging.

Authors (Year)	Task	Input Data Type	Samples Dataset	Model	Metrics
Moon et al. (33)	Evaluating CAS symptoms in TAO	Patient facial images	1020	ML-supported system	Sensitivity: 0.881, Specificity: 0.869, accuracy AUC: 0.884
Lin et al. (34)	Distinguishing between active and inactive TAO patients	MRI images	160	CNN	Accuracy: 0.86, Sensitivity: 0.82, Specificity: 0.89, F1 score: 0.71
Li et al. (35)	Evaluating inflammatory activity of extraocular muscles in TAO	Contrast-enhanced MRI images	1479	ML models	AUC: 0.926, Precision: 0.911, Recall: 0.961, F1 score: 0.936
Lee et al. (36)	Differentiating between active and inactive stages of TAO	Orbital CT images	144 active and 288 inactive TAO patients	CNN	AUC: 0.871
Zhu et al. (37)	Characterizing ocular lesions and staging TAO	TAO patient facial RGB images	2,228 facial images of TAO patients	EDWM model	Dice coefficient: 0.898, Sensitivity: 0.895, Precision: 0.931
Yao et al. (38)	Detecting inflammatory activity in TAO	SPECT/CT images	Not specified	GO-Net method	IOU: 0.82 ± 0.05%, SN: 91.01 ± 5.32%, SP: 99.96 ± 0.03%, AUC: 0.90, Diagnostic precision: 86.10%
Huang et al. (21)	Predicting TAO subtype, stage, and severity	Medical records, images	Over 1,000 medical records (953 patients)	ML models	AUROC:0.92
Lei et al. (39)	Classifying facial expressions in TAO	Facial expressions images	827 cases in dataset 1 126 cases in dataset 2	VGG-19 network	AUC: 0.847, Accuracy: 0.851, Sensitivity: 0.899, Precision: 0.899, Specificity: 0.720, F1 score: 0.899

## 4 Role of AI in TAO grading

### 4.1 TAO grading

The clinical presentations of Thyroid Eye Disease (TAO) are multifaceted. Mild cases may exhibit no subjective symptoms, while severe cases can significantly impact visual function, daily life, and even result in Corneal Ulcer, perforation, and blindness. Therefore, selecting an appropriate and efficacious treatment plan based on the degree of TAO is imperative. Currently, clinical grading methods for TAO consist of the European Group on Graves’ Orbitopathy (EUGOGO) classification and the NOSPECS classification by the American Thyroid Association, both suitable as a reference for clinical treatment planning. These classifications are also useful for clinical management. Multiple methods of assessment and grading are available, and their accurate application may require the clinician to possess some degree of experience and knowledge. Early diagnosis, identification of cases with potentially serious complications, and development of an appropriate management plan are essential (36). Diagnosing TAO in the presence of normal thyroid function can be challenging due to the wide range of clinical presentations, which can vary from unilateral dry eye disease with mild symptoms to bilateral complications that pose a threat to vision (37). Additionally, the treatment and management methods for TAO are continually progressing, and new diagnostic and therapeutic approaches are continuously emerging. Therefore, healthcare providers must keep up-to-date with the latest research and treatment options to provide optimal care for their patients (38).

AI can analyze vast amounts of data to identify and evaluate the features of Thyroid-Associated Ophthalmopathy from clinical images, CT scans, and other medical records (40). The data analysis and assessment outcomes generated by AI can provide medical professionals with valuable information to aid them in making more precise diagnostic and therapeutic decisions (39).

### 4.2 Application of AI in TAO grading

Uncertain ophthalmic features in patients with normal thyroid function or thyroiditis may impact the accuracy of grading for TAO (41). CT scans have been widely employed for TAO diagnosis. Lee et al. (42) developed a neural network model to evaluate TAO severity using 288 orbital CT images from patients with mild TAO for training and testing purposes. The model accurately differentiated patients with moderate to severe thyroid-associated ophthalmopathy (TAO) from controls, exhibiting comparable or superior performance to oculoplastic specialists. However, it demonstrated lower efficacy in distinguishing patients with mild TAO.

Moderate to severe TAO generally has a significant impact on the quality of life. Specifically, diplopia associated with enlarged extraocular muscles is challenging to treat and results in functional impairment. The activity and severity of TAO can be assessed using various methods, including the CAS and modified NOSPECS classifications. However, no comprehensive tool is currently available to assess all conditions objectively. Lee et al. (43) conducted a study involving 400 patients diagnosed with TAO and developed an automated clinical scoring algorithm using ML. This algorithm quantified the risk of developing moderate-to-severe TAO or muscle-dominant TAO and achieved better model performance than PREDIGO which was proposed by Wiersinga et al. in 2018 on all metrics except specificity (44).This indicates that it may serve as a superior instrument for TAO classification and forecasting of moderate-to-severe TAO.

## 5 Role of AI in TAO treatment and surgical decision-making

### 5.1 Treatment and surgical decision making in TAO

Treatment of TAO includes drug therapy, Orbital Radiotherapy, and surgery. Drug therapy mainly consists of treatments such as Corticosteroids, Biologics, and Traditional Immunosuppressants. As kind of drug therapy, Anti-inflammatory treatment is the primary and vital therapeutic approach for active, moderate-to-severe TAO (45). Throughout the process, risk factors need to be controlled, thyroid function needs to be maintained stable, and ocular symptomatic supportive therapy should be provided. The selection of a treatment plan for TAO must consider the staging and grading of the ailment, the therapy’s usefulness, its safety and cost, drug accessibility, and the patient’s preferences. Opting for the most effective remedy for this intricate and fluctuating condition involves significant challenges and cannot precisely anticipate the results of treatment. Hu et al. (46) combined T2WI-derived EOM radiomics with disease duration to provide a promising noninvasive method for determining treatment response in TAO patients before taking GC.

With the continued advancement of AI technology, its use in the medical field is increasingly prevalent, particularly in the realm of treatment decision-making. AI-based systems are capable of extracting and analyzing large amounts of patient data, encompassing disease progression, treatment response, and potential complications, to generate personalized treatment recommendations for medical professionals. For TAO, AI can analyze imaging data, the patient’s clinical symptoms, and results from past treatments to forecast the probable outcomes of various treatments. This assists doctors in designing the optimal treatment plan.

### 5.2 Application of AI in TAO preoperative planning

GC pulse therapy, also called Corticosteroids Intravenous pulse therapy, is the initial treatment for moderately to severely active TAO. Nonetheless, a significant percentage of patients exhibit unfavorable results post-treatment and are subjected to the negative consequences of corticosteroids.

Wang et al. (47) introduced a novel scheme to identify Graves’ ophthalmopathy patients unlikely to benefit from intravenous corticosteroid pulse therapy before dosing by developing a predictive model. This approach aims to improve precision medicine for Graves’ ophthalmopathy. They analyzed the data using clustering methods and AI algorithms to provide earlier treatment options for these patients. This study presents a novel approach to integrating AI modeling into clinical research, enabling non-AI-trained medical researchers to use the model directly. By utilizing the Artificial Intelligence Kit (AK) software, Wang et al. (48) extracted quantitative texture parameters of extraocular muscles (EOM) and orbital fat (OF), which facilitated the investigation of EOM and OF in texture analysis of MRI for monitoring and predicting the GC treatment response of TAO patients. Ultimately, the texture parameters of the extraocular muscles (EOM) and orbital fat (OF) were significantly correlated with the response of TAO patients to glucocorticoid (GC) treatment. This finding suggests that these parameters may serve as a useful tool in monitoring and predicting the response of TAO patients to GC treatment. The utilization of open-source AI software has played a crucial role in this study, enabling the researchers to obtain the data necessary for the experiment quickly and easily. Zhang et al. (49) found that the WOR model achieved satisfactory results in TED IVGC response prediction. The inclusion of more orbital structures and the use of ML algorithms would be beneficial when constructing radiomics models. The option of segmenting orbital soft tissues individually or as a whole has not yet achieved the ultimate optimal result.

Immunosuppressive therapy using intravenous steroids is a primary treatment for active TAO. Nevertheless, up to 25% of patients with moderate to severe TAO may not respond to steroids or may relapse after treatment is discontinued, and they may even experience side effects (50). Thus, forecasting patients’ steroid response is crucial. In their study, Park et al. (51) utilized XGBoost to predict steroid response in TAOTAO patients. Finally, the study determined that the presence of EOM restriction and Thyroid-Stimulating immunoglobulins level significantly impacted the ability to predict treatment response. This validating the suitability of gradient-enhanced modeling for various ophthalmic diseases.

### 5.3 Application of AI in TAO postoperative prediction

TAO orbital decompression is a surgical technique in oculoplasty that aims to prevent optic neuropathy and reduce exophthalmos. However, deciding whether or not to perform decompression surgery can be challenging due to the potential for significant changes in postoperative appearance. Yoo et al. (52) utilized a Generative Adversarial Network (GAN) to produce postoperative facial images by inputting preoperative facial images to accurately simulate the true appearance of postoperative orbital decompression surgery. The study randomly collected 1000 pre- and post-operative transformed images as inputs and tested three of the most popular GANs at the time. The results revealed that the conditional GAN model outperformed the lightweight CycleGAN, which employs a small-sized GPU. Nevertheless, standard CycleGAN can produce high-resolution and realistic images in Google’s CoLaboratory environment, albeit requiring a higher computational load to train the model. This indicates that GANs may serve as a valuable decision support tool for Orbital Decompression Surgery. However, this study is constrained by limited data, and future DL networks will require expansive datasets for training. A summary of studies related to AI in TAO treatment and surgical decision making is shown in **Table 3**.

**Table 3:** AI in TAO treatment and surgical decision-making.

Authors (Year)	Task	Input Data Type	Samples Dataset	AI Model	Metrics
Wang et al. (50)	Eyelid surgery protocol decision	Disease progression data	278	Predictive model	AUC:0.833
Wang et al. (51)	Investigating EOM and OF in MRI texture analysis for GC treatment response in TAO	MRI images	37	Artificial Intelligence Kit	AUC:0.82 Sensitivity:0.826
Park et al. (53)	Predicting steroid response in TAO patients	Clinical data	89	XGBoost	Accuracy:0.862
Yoo et al. (54)	Simulating postoperative appearance for orbital decompression surgery	Pre- and post-operative facial images	1000	GAN	AUC:0.957 Accuracy:0.909 Sensitivity:0.878 Specificity:0.939

## 6 Discussion

AI has undergone significant evolution since its conception in the 1950s and progressed from simulating basic cognitive functions to employing advanced DL techniques. In ophthalmology, DL has been utilized to analyze fundus photographs, Optical Coherence Tomography (OCT) scans, and visual fields. This has led to improved detection of various conditions, such as Diabetic Retinopathy, Retinopathy of Prematurity, Glaucomatous Edema, Macular Edema, and Age-Related Macular Degeneration(AMD) (53). The application of AI in TAO diagnosis has gradually diversified to include applications such as analysis of medical images, analysis of ocular parameters, and analysis of patient auscultation, Song et al. (20) The 3D-ResNet model was used to analyze CT images for early and accurate TAO screening. This methodology showcases the model’s accuracy, which is comparable to that of physicians under complex clinical conditions. Zhang et al. (18) utilized a DL network model to segment the ocular region in orbital CT to automatically calculate exophthalmos prominence. Researchers have proposed various AI-based techniques for staging TAO through the analysis of diverse medical images. Yao et al. (33) introduced an automated approach that uses SPECT/CT images and DL networks for identifying inflammatory activity in patients with TAO. This method underscores the value of multimodal imaging in boosting TAO staging precision, particularly when SPECT/CT imaging is leveraged, which facilitates a better grasp of the disease and improves staging accuracy. Moon et al. (29) demonstrate the innovative use of ML techniques in predicting CAS to diagnose patients in the early stages of TAO, prior to severe appearance changes. Additionally, the use of AI in TAO staging is notably innovative. Lee et al. (42) developed a neural network model optimized for diagnosing TAO and assessing its severity, which reliably distinguishes between patients with moderate-to-severe TAO and controls. Additionally, Lee et al. (43) created an automated clinical scoring algorithm using ML to predict TAO severity and type comprehensively. AI-based methods for TAO treatment decision-making are transforming clinical practice by furnishing personalized and precise options (47). A novel scheme proposed a predictive model to identify patients unlikely to benefit from Corticosteroids. Such models enable physicians to avoid ineffective treatment and associated complications and reconsider treatment options. Yoo et al. (52) utilized a GAN to create postoperative facial images through preoperative facial image inputs. This provides patients and physicians with a foundation for decision-making, ultimately lowering the chances of postoperative discontent.

Notably, Huang et al. (21) utilized a DL network to automatically segment the Eyelid Margin and Corneal Limbus, accurately measuring eyelid characteristics, while also employing DL to quantitatively assess exophthalmos. Additionally, they combined multiple AI techniques to create an integrated system capable of detecting various symptoms of TAO based on facial images. Similarly, Zhu et al. (32) analyzed 2,228 facial images of TAO patients. Using advanced DL network models, we have successfully trained an EDWM model that can precisely identify TAO. This model offers a reliable and direct diagnostic approach for the early detection of TAO and advancing CAS scores. An increasing number of studies utilize facial images for analysis due to their non-invasiveness, convenience, and affordability in contrast to other data collection methods such as CT and MRI that require expensive equipment. TAO methods based on facial images present potential as a primary research direction in the future.

Despite AI’s transformative potential in TAO management (55), challenges remain. The complexity of TAO as an ocular disease, the high cost and scarcity of data for model training, and the difficulty in generalizing DL models across diverse patient populations present significant barriers. These challenges are compounded by the ethical implications of implementing AI in clinical settings, such as ensuring patient privacy, maintaining model transparency, and managing the potential for errors.

Addressing these issues requires the establishment of collaborative networks among various centers and subjects (56, 57) to pool a wide array of datasets, ensuring a richer diversity of data. This approach would necessitate a strong emphasis on data standardization and quality control to enhance the reliability and applicability of AI models. To tackle the challenge of model generalization, adopting advanced algorithms such as transfer learning and incorporating multiple clinical variables into complex modeling techniques is crucial. Enhancing the effectiveness of AI applications also involves positioning AI as an assistive tool that supports rather than supplants the physician’s decision-making process, emphasizing AI’s role in augmenting human judgment.

In addition to technical improvements, specialized research is essential for developing more sensitive algorithms capable of identifying mild TAO cases (21), thereby overcoming one of the current diagnostic challenges. Promoting the use of AI technology in ophthalmology not only requires interdisciplinary collaboration but also a concerted effort towards public education and continuous monitoring. Such measures aim to improve the social acceptance and responsible utilization of AI, ensuring that the technology’s application in TAO diagnosis and treatment is both effective and ethically sound. Through these comprehensive strategies, AI’s role in TAO management can be significantly enhanced, leading to more accurate, personalized, and ethically responsible healthcare solutions.

## 7 Conclusion

The advancement of AI, particularly through DL has significantly impacted ophthalmology, offering refined diagnostic, staging, grading, and treatment strategies for TAO. Among various imaging analyses—fundus photographs, CT, and MRI. MRI stands out in TAO clinical management due to its direct visualization of lesions, the disease’s origin point. This precision makes MRI a focal point in AI research for TAO, enhancing diagnosis and treatment plans tailored to individual patient profiles and disease progression.

AI’s predictive capabilities extend to forecasting treatment responses and disease progression, utilizing patient data to preemptively adjust management strategies and prevent severe complications. Furthermore, AI’s role in patient self-management, through smart device integration, signifies a shift towards patient-centered care, enabling real-time health monitoring and feedback.

While acknowledging the value of multimodal data, the emphasis remains on MRI’s critical role in TAO management, underlining its unmatched value in accurately reflecting disease pathology. Future developments should prioritize enhancing AI algorithms for MRI analysis, bolstering their sensitivity to subtle clinical changes. This focus will not only improve the distinction between mild TAO cases and normal conditions but also reinforce AI as an augmentative tool in clinical decision-making, rather than a replacement.

The convergence of AI with disciplines like ophthalmology, endocrinology, and radiology offers a comprehensive approach to TAO management, suggesting a future where AI’s integration into clinical practice is seamlessly accepted and highly effective. Continuous monitoring, evaluation, and public education are essential to foster this integration, promising a future of accurate, personalized TAO treatment facilitated by AI advancements.

## Author contributions

CY: Writing – original draft, Writing – review & editing. GN: Writing – original draft, Writing – review & editing. YZ: Writing – original draft. JR: Writing – original draft. GL: Writing – review & editing. WY: Writing – review & editing. XF: Writing – review & editing.
